# Engaging with a history of counselling, spirituality and faith in Scotland: a readers' theatre script

**DOI:** 10.1080/03069885.2014.928667

**Published:** 2014-08-01

**Authors:** Alette Willis, Liz Bondi, MaryCatherine Burgess, Gavin Miller, David Fergusson

**Affiliations:** ^a^ School of Health in Social Science, University of Edinburgh, Edinburgh, UK; ^b^ School of Divinity, University of Edinburgh, Edinburgh, UK

**Keywords:** spirituality, identity, narrative approaches, pastoral care, society

## Abstract

This paper presents an abbreviated version of a verbatim script developed from oral history interviews with individuals key to the development of counselling and psychotherapy in Scotland from 1960 to 2000. Earlier versions were used in workshops with counsellors and pastoral care practitioners to share counter-narratives of counselling and to provide opportunities for conversations about historical and contemporary relationships between faith, spirituality, counselling and psychotherapy. By presenting intertwined histories in a readers' theatre script, the narrative nature of lives lived in context was respected. By bringing oral histories into virtual dialogue with each other and with contemporary practitioners, whether through workshops or through publications, the interplay between individual, institutional and societal narratives remains visible and open to change.

## Introduction

In 2010, an interdisciplinary team from the fields of theology, religious studies, literature, counselling and psychotherapy obtained funding from the Religion and Society Programme of the UK Research Councils to study the intertwined histories of religion, spirituality, counselling and psychotherapy in Scotland from 1945 to 2000. Because of the specific cultural–historical trajectory of Scotland, this history centred on the influence of Christianity, particularly the Church of Scotland, although Eastern religious and spiritual discourses also played a role, particularly Buddhism. This project involved archival work as well as the collection of oral history interviews from 18 people key to developments during the 1960s, 1970s, 1980s and 1990s.

For these oral histories, we recruited people who sat somewhere on the intersection of religion and counselling. This intersection was most often identified through their involvement in a religious institution, such as the Church of Scotland, and through their key role in the establishment and/or development of one of the early counselling services. Initial participants were asked to suggest other potential interviewees, as were experts in the fields of divinity and in counselling.

Through our archival work and the recruitment of oral history subjects, it became clear that religious organisations were integral to the foundation of counselling services in Scotland, supporting Dryden, Mearns, and Thorne's (2000) findings on counselling in the UK more broadly (Bondi, 2013).^1^ However, we did not find the clear distinction between counselling and psychotherapy delineated by Dryden et al. (2000) and Jacobs (2000). In Scotland, religious organisations were equally intertwined with more psychotherapeutically orientated work, including the formation of the Davidson Clinic, which Jacobs mentions.

Our research generated interest outside the academy, particularly amongst practitioners in counselling and pastoral care. On feedback forms, these practitioners reported being motivated to attend our day conferences because they were seeking opportunities to discuss the intersections of faith and spirituality with their own practices. Despite the role of religion in the institutional history of counselling and its importance in the lives of people key to that history, practitioners reported that these subjects were largely excluded from contemporary counselling discourses.^2^ Although discussions about religion and the history of counselling do take place, including on the pages of this journal (Dryden et al., 2000; Jacobs, 2000), the majority of counsellors we came into contact with were oblivious to such intertwined histories. The stories emerging out of our research represented a counter-narrative (Frank, 2002; Richardson, 1995) to the more medicalised and institutionalised narratives that have come to dominate counselling in Britain more recently (Dryden et al., 2000).

From a post-structuralist, narrative perspective, histories shape our present, just as the present shapes the stories we tell about our past (Besley, 2002). With this in mind, we decided to undertake a year-long knowledge exchange project to share our historical narratives and to open up further opportunities for contemporary practitioners to discuss faith and spirituality in relation to their professions and their practices.^3^ We sought to do this in a way that respected the narrative quality of our original research material and our narrative, relational understanding of people in society (Somers, 1994; White & Epston, 1990). Narrative and arts-based research methods are attracting increasing attention in the field of counselling and psychotherapy, illustrated, for example, in a recent symposium issue of this journal (Meekums, 2011). Below, we outline the particular arts-informed method we used for our knowledge exchange project: readers' theatre.

## Readers' theatre

Readers' theatre has primarily developed within education (Maher, 2006; McConaghy & Utz, 2008) and performance settings (Kaye, 1995). However, with the increased interest in arts-informed methods for academic research and knowledge exchange, the method has begun to show up in academic work. For example, academics have used readers' theatre with professionals and members of the public to raise awareness (Slade, 2012) and to stimulate discussion on taboo (Cueva, 2010) and potentially controversial topics (Savitt, 2010). This work takes an approach to knowledge exchange that sees it not as the dissemination of generalisable research findings from academics to the public, but as an ongoing dialogue between researchers, research subjects and interested members of the public in which individual, institutional and even societal narratives are discussed, critiqued and cocreated.

We began by selecting the nine oral history interviews that were richest in anecdotal material and which covered a range of experiences and spiritual and religious affiliations. Using the transcripts, we developed monologues from these interviews. The monologues ranged from 600 to 1800 words in length and were comprised of more than 99% verbatim excerpts. Developing these scripts involved significant editing, since transcripts ranged from 5000 to 26,000 words in length.

Building on the claims made for writing as a means of inquiry (Richardson & St. Pierre, 2005), we suggest that the editing undertaken was part of our ‘creative analytical process’ (p. 962). We began by identifying those portions of the interviews that were narrative in structure and which spoke to the central topic of our research, bringing these into dialogue with Connelly and Clandinin's (1999) research into the key moments in personal professional narratives. These moments included origin stories, times when personal stories came into conflict with institutional narratives and stories of exits from professions. Many of the life stories we collected were shaped by themes that interviewees returned to again and again, repeating metaphors and images. We included these phrases in the monologues. There were aesthetic considerations involved as well (Richardson & St. Pierre, 2005), with lovely turns of phrase and powerful metaphors being more likely to catch our attention and be included in the final scripts.

At initial workshops held across Scotland, we shared various sets of two or three monologues. However, this provided only a limited range of perspectives on the history we wished to present, which was often remarked upon by workshop participants. For the second wave of workshops, the first author was tasked with developing a script that would fill out this history by bringing interviewees into virtual conversation with each other.

The work done in identifying key moments, themes, images and metaphors for the monologues fed into the development of the full script. However, bringing these individual stories into dialogue with each other changed what was prioritised. While diversity of views on any topic can be easily represented in dialogue, it was difficult to find a place for topics that were only raised by one of the interviewees and so these tended to be left out. Conversely, in returning to the original transcripts with a view to creating dialogue, particular themes, events and experiences that had not seemed significant in most individual stories, emerged as central to the collective story through their recurrence across interviews. For example, it was not until work began on the dialogical script that the centrality of the Second World War to the stories of interviewees emerged. In this way, working from transcripts to monologues and then to dialogues was invaluable in terms of working with and respecting the tensions between individually lived lives and broader cultural–historical developments.

The script that follows is a slightly abbreviated version of the final one we arrived at after feedback from the workshops we conducted in 2013. The interviewees were given the opportunity to review the final script and to make any corrections. Most chose to have full attribution for their words. Names and identifying details were changed for those who chose to remain anonymous.

## The Script:

### PART 1

**Characters:** ^4^

Peter Bowes

Mary Hunter-Toner

Ken Lawson

Murray Leishman

Emily Holmes

Hamish Montgomery

Jean C. Morrison

Kathy Smith

#### Act 1 – Beginnings

**Stage Directions:** *The stage is dark. The shadowy silhouettes of 8 people sitting in a row on wooden-backed chairs are visible. As each stands up to speak, a spotlight lights them up. When each finishes, he or she sits down and the light goes off*.

Hamish: I was born in 1928 in the centre of Glasgow.

The day I was christened, was the tenth anniversary of the First World War.

The minister took me outside and held me in his arms to see the bands march past.

To this day, when I hear the words “May the Lord Bless you and Keep you …” or see a Marching Band, tears come to my eyes.

My father was very busy in the church.

And at a fairly early age I got caught up in doing a bit of Sunday School teaching.

I trained as an artist, and worked freelance all my life.

Meanwhile in church terms, I've been in the Session since the age of 22 and I'm in my 80s now, so I've been a church elder for over 60 years.

Jean: I was born in Glasgow too, in 1936.

It was a very snowy day in December.

The doctor couldn't get his car up the hill,

so I was born with the help of the nurse.

To my mother's absolute terror,

as soon as the nurse took me in her arms,

she climbed on a chair and held me up.

My mum said, “What are you doing to my baby?”

And she said, “I'm holding her up to God before she comes down to the rest of us.”

That story was reinforced in all family life:

I was to be a good girl,

I was to be devoted to the church and prayer and all the rest,

and go through all the different stages of church life as it was in those days.

That's just the way it was.

The day after I turned 21

I had one of those experiences

when I just suddenly knew something I hadn't known before.

The minister in our church said, “sometimes people give up well paid secure jobs to serve Christ in some other way”

and at that point, I knew that God wanted me to give up teaching to become a deaconess of the Church of Scotland.

Mary: I think I'm the only Catholic here.

I too was born to a fairly committed, religious family.

The religious influence was from very early years.

It was about having a relationship with God

but it was also very much about having a relationship with other people

and I think, without knowing it,

early on I got this sense of the personal Christ or the personal God being in other people.

So it was that we should be very careful and very helpful to other people.

I became a teacher, in Easterhouse^5^.

But my first thoughts were to become a social worker.

So there was always that sense of wanting to help other people.

I think all of that interest came from my background,

my Christian faith, and my family as well.

Emily: Actually, I was brought up Catholic too.

I was born to an Irish mother.

After a lot of anguish she decided she couldn't keep me

and I was adopted.

One of her requests had been that I would be adopted into a Catholic family.

My adoptive parents were converts.

They'd been searchers all their lives.

So the spiritual atmosphere of the house

was of people who were very committed Catholics

but they were quite new Catholics.

The quest for community and a sense of belonging

has always been with me.

When I was around 16, I realised it was possible to challenge some of the assumptions in the Catholic church.

But the awareness of how spirituality could be applied to the world condition,

to the larger suffering, stayed with me.

When I came to university,

it got me right in the centre of faith and social action

through the Catholic Chaplaincy here in Edinburgh.

Ken: My father was a Church of Scotland minister, very conservative.

Throughout my childhood,

I desperately wanted to get his attention.

I think I got God and father mixed up

because he had the authority

And by Jove he used it

I had polio.

Went to a kid's hospital.

Great fun.

I remember being quite sad to go home.

Hospital was an insight.

It was a community to which I belonged

because I walked with a limp.

I went to New College to train to be a minister myself.

We were all new boys together in the residence

all studying to be ministers

That was a real good community.

But there was an underground something going on in that period.

There was a kind of battle I suppose

between the old way and whatever the new way was to be.

Peter: I was brought up virtually without my father.

He was a Japanese prisoner of war

missing from when I was 1 to when I was 10 and a half.

He died when I was 18.

Not surprisingly, I left home at 17 to join the army,

I became a regular soldier for 3 years.

That's what my father was, a regular soldier.

As a soldier, I went to a dance hall in Manchester and met Maureen.

She had become an Evangelical Christian and a Baptist at age 16.

I used to sit outside the chapel on a Sunday afternoon and wait for her to come.

Wise people that they were in that little chapel,

they said “why don't you come in and wait, don't sit in the cold.”

And I found a whole group of people, who were doing something similar to what I was doing,

just trying to make a meaning out of life without actually calling it that.

Murray: I was born in 1931.

When I was 9, I was walking along in the Pentlands^6^.

There was a bang and I turned

There was a boy in bits, literally.

I discovered later he had picked up a mortar bomb.

And it began to really bite into me.

I was quite, you'd say, disturbed

with nightmares and delusions.

Everybody said, “what on earth is wrong with this kid?

Nobody knew.

At 13, I had a religious conversion.

I was received into the church and became a member of a very lively youth club,

part of post war reconstruction.

These youth club leaders were coming back from the front.

Men who had been through pretty horrible things themselves

and who had a pretty good idea of what us kids needed.

I went to New College and eventually became a minister.

I had a lot of fun,

learned a lot,

preached too much.

Found myself more and more reading psychology,

and thinking, “what a terrible heathen I am,

I should be reading St John's Gospel etc, etc.”

So there was all this fight in me about that.

Kathy: I was born just after the war

in a small village in England.

My mother would regard herself as agnostic.

My father was atheist, almost devoutly so.

They brought us up in the spirit of questioning and one could say non-conformity with a small n and a small c.

I wasn't baptised, deliberately so.

And I think I regarded that status with something of pride.

Most of the children I went to school with would have gone to church and would have described themselves I suppose as C of E.

So I grew up with a lot of features of outsider-ness in my upbringing.

Soon after I went to university

I found the community I was looking for in the Unitarian Church.

I found it a remarkable place,

tolerant of scepticism and sceptics,

people who didn't otherwise conform.

So I joined in with them and that was very enjoyable

and I felt a sense of belonging

which I think I had probably been searching for for a long time,

which I wouldn't put down to having found necessarily so much a spiritual home

as a group of like-minded people who were welcoming and accepting.

#### Act 2 – Individual Challenges

Peter: I discovered there was no way I could be a regular soldier.

I was impossible to discipline.

So I left and studied to be an engineer.

Got a job and eventually moved to Hamilton^7^.

My wife and I became part of the Baptist scene there

which is fundamentalist, conservative and more exclusive than I'm ever going to be comfortable with

Yet seven of us went into Baptist ministry,

which is quite astonishing,

and I was one of them.

At the time I would have told this in terms of a call to the ministry.

But now as a psychologist,

I would much rather think about in terms of a typical Type A personality under pressure and stress

needing to find a way out.

So I jumped ship.

Ken: For me, it was being a minister that was stressful.

My second parish was in Cumbernauld^8^.

Great people from all over the place

all thrown together

in a place that had little sense of community.

I never knew who was behind a door I knocked on.

They could be any kind of background

and that was so refreshing and scary.

I think the child in me rebelled

and said “I don't know where I am anymore”.

I was very challenged, because there was no party line.

Within two years I had a collapse.

Emily: I was working as a social worker

when in 1975, my mother died suddenly.

That propelled me into psychotherapy for myself.

Because of the dreams and the nightmares.

Jean: As a deaconess, the biggest challenge was pastoral care.

The only training I had was that the lecturer at college,

took us through the Gospel of Mark

and showed us how Jesus had responded to people

then told us to go out with the Bible and prayer

and be with people the way Jesus had been with people.

I remember sitting in somebody's living room,

on a winter afternoon as the room darkened

and she began to talk about experiences of her dead husband coming back.

I was scared shitless.

I didn't know how to pray in situations like that.

I didn't know how to read the Bible to them in situations like that.

People who were mentally ill,

I didn't know how to relate to them.

People who had a drink problem,

I had been brought up to be teetotal,

all I could do, was to tell them that they should really try to get off the stuff.

I hadn't the foggiest.

I felt so de-skilled.

Hamish: When I trained for the office of Reader at my church,

there was no kind of training in pastoral work at all.

I spoke to my minister

and he said that he had been at a course with Dr Frank Lake^9^

and he said, “I think you would benefit greatly from meeting him”.

So that then took me in that direction.

Kathy: When my husband and I had children,

I went to the National Childbirth Trust classes,

the popularity of which I'm sure has a lot to do with the fact that women found themselves geographically separated from their family.

And because this group had been very supportive to me

I became very interested in supporting other mothers with breastfeeding and Breastfeeding Counselling.

And the counselling that I did spilled over,

as I can readily understand now why it should,

from supporting women with breastfeeding

to hearing about their experiences of depression

and their experiences of marital disharmony,

their experiences of sudden infant death

or of children who were ill or with disabilities.

Or illness in the women themselves,

which could be life threatening such as breast cancer.

The training I'd been given didn't cover that.

Murray: The most dangerous youth club I ever ran was in Glasgow

The boys said to me one day,

“you'd better talk to Jimmy, he's aff his heid.”

So I thought, well, what's that?

And I leaned against the wall -

you never spoke straight at people,

you leaned against the wall –

and said, “how are you doing then?”

Jimmy replied, “my faither came doon the stair the other morning and said, your mother's deid, dinnae greet.” [don't cry].

And suddenly I was turning on sufficiently to know, this is a very disturbed boy.

What do you do with that?

How do you respond to that?

And he was acting it out in very strange and violent ways.

Behind that violent behaviour,

something violent had happened to him

which could not be spoken of or thought about.

These pointers kept appearing in my life as a minister.

I thought, I've gotta get hold of some basic facts of what it is that enables boys and girls to grow up into good men and women

because we don't know.

And then I knew that I'd made a mistake.

What was needed was not more religion

but more psychologically informed faith.

So after 5 years, I started packing my bags.

### PART 2

#### Act 3 – Doors Opening

**Stage directions:** *the curtain opens to reveal the same 8 people sitting on wooden chairs in a semi-circle. They are facing each other and the audience. The light is on them all.*

Emily: After my mother died,

I went to a therapist at the Scottish Institute of Human Relations^10^.

She then recommended I go and see Dr Winifred Rushforth^11^.

Winifred was like the grandmother that I hadn't had.

There was that kind of forthright, apparently uncompromising, straight-gazed, no-non-sense woman,

but who contained a tremendous ability to listen and to understand

and to be unshocked by anything really.

She said that she would like me to become a member of one of her dream groups,

but only when I had been to her Sempervivum Easter School, in the Borders.^12^

The idea behind it was to integrate the insights of religion with those of psychotherapy.

Winifred had a grounding in both.

So I went off to the Sempervivum Easter School

and found it an extraordinarily rich place to be.

It was overwhelming and scary

and a real opening into the possibilities

of shifting consciousness,

which became a significant turning point in my life.

I think what is important to remember now

when counselling and psychotherapy has expanded so hugely,

is that back then there was no Counselling training, with a capital C, in Scotland.

Peter: I got my initial training at the Southern Baptist Missionary Seminary in Switzerland,

where I studied for 4 years, full-time.

I learned a lot from the pastoral care and counselling side of the teaching there.

The training came from a North American Evangelical movement,

not in its fundamentalist form,

but in its profound social gospel form.

I thoroughly enjoyed it.

Jean: There's an American connection for me too.

I was awarded a scholarship to Chicago Theological Seminary,

and there were courses in pastoral psychology,

and I thought, “gosh, this is what I'm wanting.”

It was the beginning of my exposure to the whole counselling world.

I must have spent most of that year with my mouth open in awe.

I'd no idea people could be so skilled,

could be so compassionate.

I had just been full of fear with the people who needed me.

When I realised there was training I could do,

that really launched me off.

Murray: My biggest influence was Jock Sutherland.

When he came back to Scotland,

the door really swung open.

Jock was an extraordinary chap.

He took me on as a kind of apprentice,

I learned more in the process of osmosis and identification with him

than I did from the 4-years of formal training I took.

Eventually there were 8 of us.

We became the Scottish Institute of Human Relations.

Hamish: My wife and I did the 2 years of seminars with Frank Lake in clinical theology.

At the end of it, Frank said, “would you like to do another course and become a tutor?”

So we both then did that

and tutored for the Clinical Theology Association for about 20 years.

It's called the Bridge Pastoral Foundation now

and it celebrated its 50^th^ anniversary last year.

Mary: I came to counselling later than most of you,

in the 1980s.

Around the time I started teaching,

I read in our church bulletin

that there was this organisation called, the Catholic Marriage Advisory Council,

People who might be interested in becoming counsellors were invited to come along to an open meeting in the diocese.

So I did.

I hugely enjoyed my training as a counsellor,

but found it hugely challenging.

I reckon that was the period where I as an individual grew the most in my life.

Ken: I mentioned my break-down, right?

Well after that, the old patterns just collapsed.

There was no tradition to fall back on.

It was a huge turning point.

I went to this group at the Southern General.

The psychiatrist who led it was very much a facilitator, drawing stuff out of us

saying, “go away and think about it.”

And that's what I recognised as being therapeutic.

I felt listened to and understood and challenged

and I thought “there's something going on here.”

I thought, “my goodness, this is helping.”

And ministry came into that: “this is a kind of ministry this guy's at.”

“How do I integrate this kind of healing process into my own ministry?”

Back in Cumbernauld, three of us formed a real strong ministry team.

Hamish ran this 2-year clinical theology course.

And the great thing was, the three of us went to that.

That was totally life changing.

We put into practice what we were learning together,

learning to be open with one another.

Somewhere around that time,

Transactional Analysis^13^ became a thing in the Church of Scotland.

I first learned about it with Hamish,

but I went on to study with Jean.

All this was part of my ministry

but it was also part of my own therapy,

cos I was learning mightily along the road.

I was having insights into connections, ministry, therapy and small groups.

#### Act 4 – Institutional Challenges

Jean: When I got back, the Church of Scotland had made a new job for me,

working with Archie Mills, who was Director of Counselling, Development and Training.

We'd both gone to an introductory course in Transactional Analysis,

TA for short,

and as we talked we said,

“this is a wonderful tool for us to use to help people understand how to be a Christian.

It's a secular tool,

but it's as if it's a fulfilling of Jesus' command: love your neighbour as you love yourself.”

So we asked permission to take professional qualifications in TA.

In the 70s, the Church of Scotland was creative and open to new things.

One of the big theories of TA is, “I'm OK, you're OK.”

Now, that was confronted hugely by people who said, “that doesn't fit with our theology because, of course

we're not OK.

We are people with original sin,

so how can you possibly say, I'm OK, you're OK?

I'm a sinner, you're a sinner.

And you live with that.

And of course, through Jesus,

he sees us as perfect

and God sees us as perfect through Jesus.”

That's the theology.

We tried to explain that what we saw in the Gospels

was the same as we were understanding in teaching TA.

We would say, “this is the way Jesus behaved with people.

He believed in them.

If he said, go and sin no more,

he believed in them enough to believe that they would

at least try and be better.”

But some people said,

“well of course, Archie and Jean are just preaching the gospel of TA,

not the gospel of Jesus.”

Hamish: (Snorts)

I encountered some of that same attitude when I took over as Director of the Tom Allan Centre.

At the time, the Centre was concerned with single homelessness.

I thought, well that's fair enough

but if we can get them before they get there,

it's infinitely better.

So I set about developing it as a counselling centre.

At that time there were very few counselling centres about.

Some were a wee bit opposed to it,

particularly those who were a wee bit further over to the right.

There was a suspicion of quotes “psychological stuff,”

and there was that very strong feeling with some that prayer was the only answer.

I'm sure prayer is the answer in many situations,

but it seemed kind of blinkered.

It would be like forgetting anaesthetics had been invented,

if you were ignoring all that had happened in the field of psychology.

Peter: That's exactly why we organized the Pastoral Foundation in Edinburgh the way we did.

We set it up to try and avoid these politics.

It started out as an idea that an ecumenical group of ministers had.

We had a vision of what was actually needed.

We didn't buy into the “oughtness” of what the evangelical tradition and Presbyterian Church thought should happen.

We set it up as a separate charity

I was minister of Morningside^14^ Baptist at the time.

What the Baptist church wanted to know,

is how many people we converted and how much it would cost.

You just don't answer questions like that.

The PF represents a way in which the national church politics could be side-stepped.

It would never have survived as a church venture.

I'm very pleased it's still there.

Mary: I understand where you're coming from, Peter.

But I do think it's possible for a closer relationship between churches and counselling services.

I took over Catholic Marriage Care in 1999 as chief exec.

We had income from the government and from the church.

We had to be sure that we were serving the whole community.

I had a real sense that having Catholic in our title put people off.

So we became Scottish Marriage Care.

The hierarchy within the church,

even some priests on the ground,

they want some sense that we're still linked to them.

And my line has always been,

“We are linked to you,

we do what I see as Christian work,

that's what I do.

Although other people maybe do it from a different perspective.”

And what we have to help them understand,

is that a counselling organisation and a religious organisation can work alongside each other,

but they don't always meet on every level.

Emily: Religion, spirituality and counselling can even enrich each other.

From Samye Ling, the Tibetan Buddhist Monastery^15^, into Edinburgh came the Tara Trust,

which was a therapy centre run by Buddhist-trained therapists.

And I got involved.

It seemed that the Tara Trust had a finely tuned combination of spirituality and therapy

that was a very powerful and very instructive model for people.

#### Act 5 – Passing the Baton

**Stage Directions:** *The lights go down and the actors move their chairs back into a row. As each character stands up to say their piece, the spotlight finds them and fades to black as they sit down again.*

Jean: This is a truism, but God knows me so much better than I know myself –

I have been conscious of being led through life sometimes

so that I was carrying out my calling to be a deaconess,

to serve Him,

working in the community, working in the church

even in retirement.

My life sort of makes sense to me looking back.

Hamish: The religion or the psychotherapy or the art

it's all wrapped up – in me anyway.

It's all the one thing.

Peter: At best, if I ever was anything, I'm a Christian who does counselling

and can offer a pretty well worked out philosophy of what counselling is.

To my mind, what I do in the counselling room,

if those values were expressed in the community as a whole,

the Kingdom of God would come.

Mary: I think the gap between clergy and people has very much narrowed.

Lay people have a much more prominent role in terms of spirituality,

in terms of doing that bit which I would do, and have done, and people still do,

which is doing the thing that Christ did,

which was to serve others.

Murray: I got to a point where religion was like a tired old horse that was dying under me.

Being a minister and being involved in people's family lives and so on,

you realise that what you're really viewing are some of the great tragedies of the world.

And what I think distresses all of us

is the tiny grasp of reality that we sometimes have in the church,

in our tiny little services and so on.

To think of the people who are struggling away,

human beings like me, like you.

To really try to get to what it must be like to be this person,

that to my mind is the first and foremost question.

And I suppose psychotherapy seemed to offer more opportunity to express that

and to explore that

than the church did

Ken: For me now,

spirituality is that which holds everything else together.

It's at the root of all.

Sometimes I wonder

what on earth I'm doing here.

There must be more than this.

And that's where I still am.

I believe there is more,

but don't ask me what

and don't tell me

in little packages

what it is.

Kathy: I was wondering whether one of the reasons why I'm a psychotherapist

is because perhaps one of my desires is that psychotherapy will help to rehabilitate the outsider,

bring the non-conformist or the non-believer in from the cold

and give them salvation.

Is that what I'm seeking?

It's a troubling thought!

Emily: I think both therapy and spirituality are needed.

People are wanting and needing something that gives meaning in their lives,

that allows them to contemplate the bigger than themselves,

bigger than the I,

bigger than the ego,

bigger than the individual.

And I think only when therapeutic ideas become less individualistically orientated and incorporate ideas and philosophies of being;

and only when religions become less dry and narrow and take on all the wisdom and ideas and theories of human development;

only when the two combine and greet each other and work together can we have a working system of understanding how we can go forward in this 21^st^ century.

Coda: We do not present this script as the definitive history of counselling in Scotland, but rather as a partial, local history emerging out of the storied experiences of a set of key people refracted through our own creative analytical processes. We offer the script as a narrative resource in the ongoing, collective storying of our field. Readers' theatre enables research to be communicated in a way that represents the scaled nature of narratives present in society and the impacts narratives operating at these different scales have upon each other. While demonstrating that individual stories clearly draw upon and are shaped by broader institutional and social narratives, our script invites audiences in to witness the ways in which people living storied lives can challenge and ultimately impact upon institutional narratives and upon history itself.

## Notes

1. Bondi (2013) provides an overview of the role religious organisations and discourses played in the formation of key services in Scotland. See also Miller (2008).

2. Leseho (2007) makes a similar point about counselling and counselling training in Canada.

3. The response of these contemporary practitioners is discussed elsewhere (Willis, in progress).

4. For the purposes of review, all the interviewees have been given pseudonyms. Some interviewees asked for full attribution and will be listed as characters under their actual names when this article is published. Others asked to be anonymised and will continue to be given a pseudonym as well as having identifying details altered.

5. Easterhouse is a large housing estate on the eastern periphery of Glasgow built in the 1950s and 1960s to rehouse working-class families from cramped, substandard tenement dwellings in the city. It remains one of the poorest and most disadvantaged neighbourhoods in Scotland.

6. A range of hills near Edinburgh.

7. A town near Glasgow.

8. A new town in central Scotland built in the late 1950s and early 1960s to rehouse poor working-class families from slum dwellings in Glasgow. Its modernist, brutalist architecture combined with social deprivation earned it the reputation as one of the worst places to live in Scotland.

9. Frank Lake founded the Clinical Theology Association in 1962. The association provided training to clergy to support their pastoral work and to promulgate an understanding of distress informed by psychology and psychotherapy.

10. The Scottish Institute for Human Relations was founded in 1971 and became the centre for psychoanalytic training in Scotland.

11. Winifred Rushforth (1885–1983) worked as a medical missionary in India for 20 years and went on to develop community-based psychotherapy in Edinburgh in the 1940s. During the 1960s and 1970s, she pioneered the blending of eastern religious ideas (to which she had been exposed in India) with Western psychotherapy (Miller, under review).

12. A rural region of Scotland, south of Glasgow and Edinburgh and immediately north of the border with England.

13. Ken, Hamish and Jean were involved in the adoption of Transactional Analysis as therapeutic orientation within services developed by the Church of Scotland. During this period, psychodynamic approaches were also influential (especially via the Scottish Institute of Human Relations). Person-centred, transpersonal, gestalt and integrative orientations were also taken up in the developments to which our oral history interviewees contributed.

14. A popular, broadly middle-class neighbourhood in Edinburgh.

15. Located in the Scottish Borders.
